# Rapid cyclic ion mobility separations of monosaccharide building blocks as a first step toward a high-throughput reaction screening platform for carbohydrate syntheses†

**DOI:** 10.1039/d1ra08746k

**Published:** 2021-12-14

**Authors:** Tyler L. Peterson, Gabe Nagy

**Affiliations:** Department of Chemistry, University of Utah 315 South 1400 East, Room 2020 Salt Lake City Utah 84112 USA gabe.nagy@utah.edu

## Abstract

Herein we present a new high-throughput screening method for carbohydrate syntheses based on cyclic ion mobility spectrometry-mass spectrometry (cIMS-MS)-based separations. We rapidly resolved the α/β anomers for carbohydrates with varying protecting groups after only 5 m of cIMS-MS separation and also detected their respective unwanted anomeric impurities at levels lower than 2%. All experiments were performed in 1 minute of total acquisition time demonstrating our method's high-throughput nature. Our methodology was also extended to the separation of an isomeric mixtures of two protected disaccharides illustrating its utility beyond only monosaccharides. We envision our presented workflow as a first step toward the development of a high-throughput screening platform for the rapid and sensitive detection of α/β anomeric selectivities and for trace isomeric/isobaric impurities.

## Abstract 2

High-resolution cyclic ion mobility separations enable the rapid resolution of α/β anomers in carbohydrate building blocks and their associated anomeric impurities.

## Graphical abstract

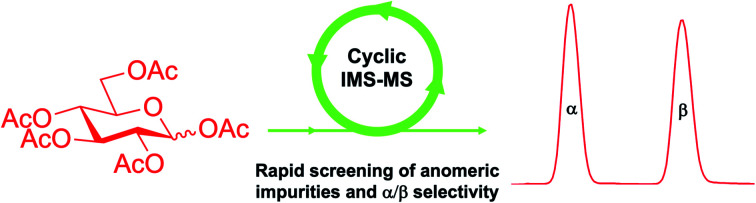

## Introduction

It goes without saying how important carbohydrates are in various biological processes (*e.g.*, cellular functions, post-translational modifications, regulating immune responses, *etc.*).^1–5^ As compared to other biomolecules, carbohydrates undergo non-template driven syntheses resulting in a tremendous degree of isomeric heterogeneity due to their various glycosidic linkages, α/β anomers, and linear/branched chains.^3,5–8^ While bioanalytical techniques have made great strides in recent years to better characterize carbohydrates of interest, further progress has been hindered by the lack of authentic standards currently accessible through synthetic methods.^9–12^ Even with advancements in automated synthesis platforms,^13,14^ synthetic schemes remain difficult and laborious due to the numerous protection/deprotection steps required to synthesize a final product based on the wide reaction landscape needed to be assessed (*e.g.*, desired final purity and anomeric selectivity, concentrations, types of solvents to be used, temperature, pH, *etc.*).^15–19^ Thus, high-throughput screening (HTS) platforms that can enable all of these conditions to be surveyed in a rapid fashion are desperately needed to further advance the field of glycobiology. However, this remains a difficult analytical challenge since reaction products from carbohydrate syntheses will predominantly be isomeric in nature stemming from their α/β anomeric mixtures as well as the possibility for regioisomeric species after potential protection group migration.^10,19–23^ Furthermore, existing analytical methodologies still largely remain blind to identifying even minor unwanted reaction side products (*i.e.*, ≤5%) which preclude the ability to confidently scale up synthetic schemes to gram-scale quantities.^24–26^

Mass spectrometry (MS)-based approaches have dominated the HTS field for monitoring the reaction products of peptides or other small molecules because of its ability to easily, and rapidly, resolve nonisobaric species from one another (*i.e.*, final product from starting material and/or intermediates).^27–32^ Unfortunately, since anomeric mixtures or other isomeric impurities have identical molecular weights, MS, alone, cannot be used for the HTS of carbohydrate syntheses. Nuclear magnetic resonance (NMR)-based approaches can definitively identify the structure of a highly pure carbohydrate reaction product, but requires milligram-level quantities, are slow in nature (*i.e.*, up to several hours), and struggle to detect minor anomeric impurities (<5%).^24,25^ All of these factors essentially eliminate NMR from contention as the analytical method of choice for HTS of carbohydrate reactions. Alternate-pump recycling liquid chromatography (rLC)^33–35^ has demonstrated purification of building blocks from their unwanted impurities,^36^ but its lengthy timescale (retention times on the order of 10s of minutes up to hours) prevents the rapid screening of multiple reaction conditions or aid in the optimization of existing workflows on a desired timescale of seconds. While we envision that alternate-pump recycling liquid chromatography can be used as the final purification step of a desired reaction product, we are, instead, interested in developing HTS methodologies that can rapidly and sensitively identify reaction products, especially those stemming from minor anomeric impurities. Ion mobility spectrometry coupled to mass spectrometry (IMS-MS), where ions separate in the gas-phase under the presence of an electric field based on their size/shape (mobility) and charge has shown potential to rapidly (on the order of milliseconds) separate out certain isomeric species,^37,38^ and thus could form the foundation of a HTS platform. Unfortunately, poor IMS resolving power has limited this opportunity from coming to fruition. We would like to mention that a previous report indicated that a 25 cm IMS-MS platform was able to resolve some synthetic carbohydrate isomers, albeit ones with long alkyl chains at the anomeric position that would lead to greater expected mobility differences.^39^ Recent advances in IMS technology have increased achievable resolving power,^40,41^ but it remains unclear if anomers of commonly used synthetic building blocks can even be resolved because of their very similar structures and if minor anomeric impurities (<5%) can be detectable *via* IMS-MS. Herein, we assessed the utility of a high-resolution cyclic ion mobility spectrometry-mass spectrometry (cIMS-MS) platform for its potential to rapidly and sensitively separate out the α/β anomers of common monosaccharide protected building blocks. We envision our presented methodology as a potential first step toward the development of a HTS platform for synthetic carbohydrate reactions as well as for the rapid purity screening of anomeric impurities.

## Experimental

A cyclic ion mobility spectrometry-mass spectrometry (cIMS-MS) platform, which is commercially available (Waters Corporation, Wilmslow UK), was used for all experiments.^40^ Ions were generated through electrospray ionization (3 kV) operated in positive ion mode at a flow rate of 1 mL min^−1^. The [M + Na]^+^ adduct (which was the most intense precursor ion in all cases) was *m*/*z* selected with a quadrupole before undergoing traveling wave-based IMS separations in nitrogen buffer gas (1.74 mBar). For each experiment, ions were subjected to 5 total cycles (5 meters of cIMS separation) and then routed to the time-of-flight mass spectrometer operated in ‘V’ mode for detection. MassLynx and Quartz software were utilized for all data acquisition and processing. Signal averaging was performed for 1 minute for all experiments and no additional smoothing was performed. Protected carbohydrate standards were purchased from Sigma-Aldrich (Milwaukee, WI USA), Santa Cruz Biotechnology (Dallas, TX USA), and Carbosynth (Berkshire, UK). For purity levels provided by each manufacturer, see Table 1. Stock solutions were prepared at concentrations of 10 mM in 100% methanol. Samples subjected to direct infusion were prepared at concentrations of 5 μM in 49.75/49.75/0.5 (v/v) water/methanol/acetic acid, with the exceptions of 2,3,4,6-tetra-*O*-acetyl-α/β-d-glucopyranosyl trichloroacetimidate and ethyl 2,3,4,6-tetra-*O*-acetyl-α/β-d-thioglucopyranoside, which were in 50/50 (v/v) water/methanol. We note that the addition of acetic acid caused apparent hydrolysis in these two instances, as observed by no precursor ions being detected and the fragment ion corresponding to the loss of the protecting group at the C1 OH being the base peak observed in the mass spectrum.

**Table 1:** Protected carbohydrates analyzed and their associated purities provided from commercial vendors

Analyte	Purity
α-d-Glucose pentaacetate	≥99%
β-d-Glucose pentaacetate	≥98%
α-d-Glucosamine pentaacetate	≥99.2%
β-d-Glucosamine pentaacetate	≥97%
α-d-Galactose pentaacetate	≥98%
β-d-Galactose pentaacetate	≥99.61%
Methyl 2,3,4,6-tetra-*O*-acetyl-α-d-glucopyranoside	≥95%
Methyl 2,3,4,6-tetra-*O*-acetyl-β-d-glucopyranoside	≥95%
2,3,4,6-Tetra-*O*-acetyl-α-d-glucopyranosyl trichloroacetimidate	≥97%
2,3,4,6-Tetra-*O*-acetyl-β-d-glucopyranosyl trichloroacetimidate	≥98%
Ethyl 2,3,4,6-tetra-*O*-acetyl-α-d-thioglucopyranoside	≥98%
Ethyl 2,3,4,6-tetra-*O*-acetyl-β-d-thioglucopyranoside	≥98%
4-Nitrophenyl α-d-galactopyranoside	≥99%
4-Nitrophenyl β-d-galactopyranoside	≥98%
Sucrose octaacetate	≥99%
α-d-Cellobiose octaacetate	≥98%

## Results and discussion

**Fig. 1:**
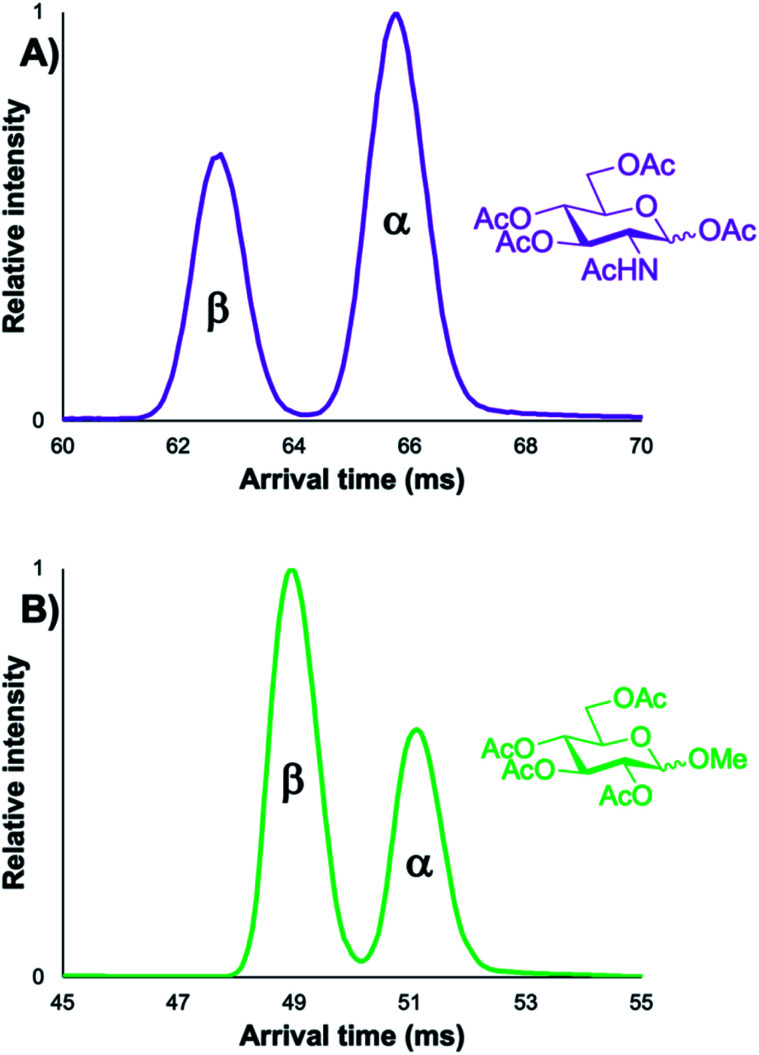
5 m cIMS-MS separations of an equimolar mixture of α/β-d-glucosamine pentaacetate as their [M + Na]^+^ adducts at traveling wave conditions of 375 m s^−1^ and 17 V (A) and 5 m cIMS-MS separations of an equimolar mixture of methyl 2,3,4,6-tetra-*O*-acetyl-α/β-d-glucopyranoside as their [M + Na]^+^ adducts at traveling wave conditions of 450 m s^−1^ and 20 V (B).

As previously mentioned, initially it was unclear whether the α/β anomers of protected carbohydrate building blocks could even be resolved with cIMS-MS given their high degree of structural similarity. For example, previous literature has demonstrated that the α/β anomers for unprotected (*i.e.*, free) carbohydrates could only be separated after lengthy ion mobility separations (on the order of 10s of meters);^42^ in some cases, these anomers could not be resolved whatsoever.^21^ In other IMS-MS-based studies, certain carbohydrate anomers chemically modified with a lengthy alkyl-chain linker to increase their structural differences were able to be resolved.^39^ However, no previous reports have assessed whether ion mobility separations could rapidly resolve out various α/β carbohydrate anomers (*i.e.*, ones with varying functional groups at the anomeric position), let alone assess the utility of such separations to detect minor, inherent, anomeric impurities. Thus, in order to test if a cyclic ion mobility spectrometry-mass spectrometry (cIMS-MS)-based workflow would be viable for the rapid and sensitive separation of protected carbohydrates, we first analyzed two different pairs of α/β monosaccharide building blocks (Fig. 1). These protected carbohydrate building blocks shared acetyl groups at the C-2, 3, 4, 6 positions, but differed in their respective protecting groups at the C-1 position (*i.e.*, methyl *versus* acetyl). Fig. 1 illustrates that both pairs of α/β protected monosaccharide building block anomers were fully resolved after 5 m of cIMS separation with a total acquisition time of 1 minute. Furthermore, it was observed that the β-anomer was higher in mobility (*i.e.*, faster) than its α-anomer counterpart for both species, potentially indicating a diagnostic mobility fingerprint trend. We note that although these compounds were run as equimolar mixtures, varying ionization efficiencies and/or presence of non-isobaric impurities could account for the differences in their respective peak areas. Based on our ability to resolve these protected carbohydrate anomers, we next turned our attention to assess whether: (1) such separations would be possible for compounds with other protecting groups and with other monosaccharide constituents, (2) the trend of the β-anomer arriving before the α-one would hold true, and (3) we could sensitively separate out minor anomeric impurities (*e.g.*, ≤5%).

**Fig. 2:**
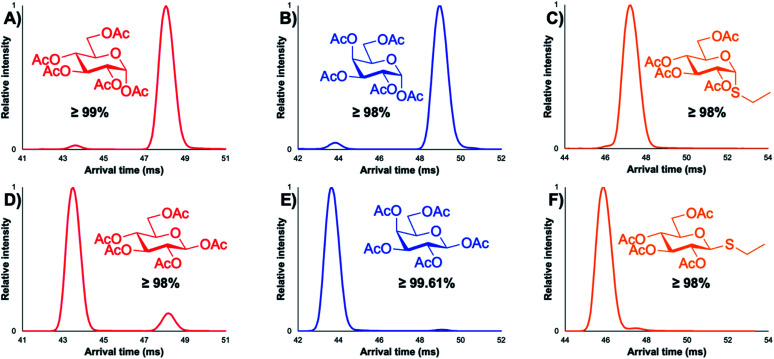
5 m cIMS-MS separations of individual protected carbohydrate anomers as their [M + Na]^+^ adducts. (A and D): α/β-d-glucose-pentaacetate at traveling wave conditions of 450 m s^−1^ and 22 V. (B and E): α/β-d-galactose-pentaacetate at traveling wave conditions of 450 m s^−1^ and 22 V. (C and F): ethyl 2,3,4,6-tetra-*O*-acetyl-α/β-d-thioglucopyranoside at traveling wave conditions of 450 m s^−1^ and 25 V.

**Fig. 3:**
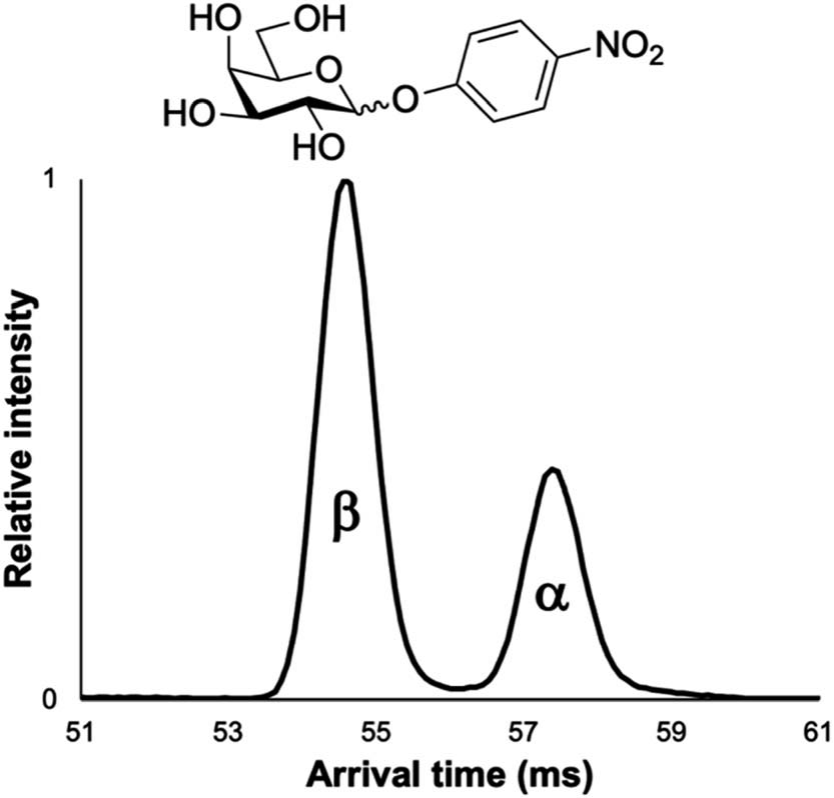
5 m cIMS-MS separations of an equimolar mixture of 4-nitrophenyl α/β-d-galactopyranoside as their [M + Na]^+^ adducts at traveling wave conditions of 500 m s^−1^ and 20 V.

**Fig. 4:**
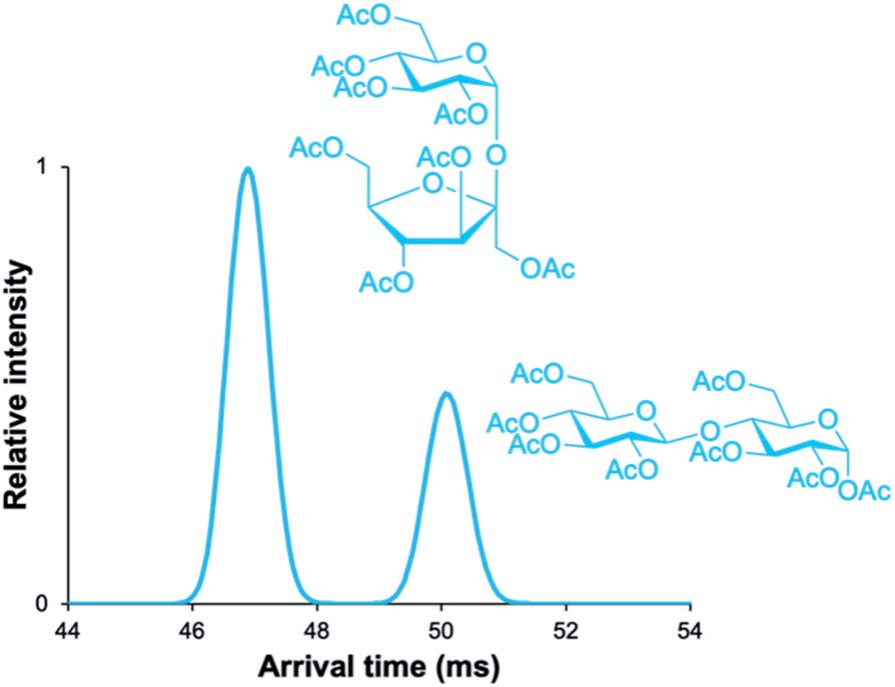
5 m cIMS-MS separations of an equimolar mixture of sucrose octaacetate and α-d-cellobiose octaacetate as their [M + Na]^+^ adducts at traveling wave conditions of 350 m s^−1^ and 25 V.

While all three of the aforementioned questions we wished to answer are relevant to this presented work, we were most curious to test if minor anomeric impurities could be resolved with our presented cIMS-MS methodology. We hypothesized that even though the listed purity levels from the commercial vendors were quite high (listed as ≥98% in all cases; see associated figure captions and the ESI†), we might still be able to separate out and detect very minor anomeric impurities for various protected monosaccharide building blocks. If successful, this would enable a cIMS-MS-based HTS platform for assessing anomeric selectivity and thus expedite reaction optimization in existing synthetic carbohydrate chemistry workflows. Thus, in order to test this, we analyzed protected monosaccharide building block anomers individually for the presence of their unwanted anomeric impurities (Fig. 2). We stress that no sample carryover occurred as ensured by thorough rinsing between experiments and also utilizing blanks. From Fig. 2, it is observed that our presented cIMS-MS-based separations enabled the highly sensitive detection and rapid separation of minor anomeric impurities in monosaccharide building blocks. We note that peak assignments were not only based on the individual runs from Fig. 2, but also from equimolar mixtures (see ESI†). Based on our demonstrated separations, it is evident that building blocks with varying protecting groups and varying monosaccharide constituents can be readily resolved in 1 minute of total acquisition time and after only 5 m of cIMS separation. Furthermore, from Fig. 1 and 2, we observed a global trend: the β-anomer always arrived earlier than the a one. We envision that this result could facilitate the rapid identification of anomeric impurities where authentic standards are unavailable; further work is needed to determine if this anomeric arrival time order trend would also apply to protected oligosaccharide species. In analyzing what type of protecting groups enabled the highest cIMS resolution of these α/β anomers, it was observed that smaller-sized ones (*i.e.*, methyl *versus* acetyl at the C-1 position) led to poorer separation (see Fig. 1B*versus*Fig. 2A and D). Interestingly, the thioethyl protecting group (Fig. 2C and F) resulted in even worse resolution compared to methyl/acetyl groups. Clearly other thiol-based protecting groups need to be studied to determine if this observation would hold true. While our results from Fig. 1 and 2 demonstrate the utility of our presented methodology, we were also interested in assessing its broad utility for other monosaccharides without acetate protecting groups at the 2, 3, 4, and 6 positions as well as for protected oligosaccharides. For these reasons, we first chose to analyze the α/β anomers for 4-nitrophenyl galactopyranoside, which is a common benchmark enzyme substrate for galactosidases.^43^ This set of anomers only contains a 4-nitrophenyl functional group at the anomeric position, with the remaining hydroxyl groups unprotected. It was unclear whether these anomers would even separate given that our previously analyzed protected monosaccharides (Fig. 1 and 2) contained mostly acetate-based protecting groups. From Fig. 3, it is seen that these nitrophenyl-protected monosaccharides were well resolved after 5 m of cIMS separation, thus demonstrating the broader utility of our presented workflow for the separation of α/β anomers for a wide array of protected monosaccharides. Additionally, we note that the arrival time order trend of the β-anomer arriving earlier than its α-counterpart also held true for these nitrophenyl-modified compounds thus implying this trend is more universal than just for acetate-based protecting groups. Since not all synthesized carbohydrate products will exist as a single isomer/anomer, we also were interested in assessing our cIMS-MS-based methodology for the separation of two fully acetylated disaccharide isomers. In Fig. 4, it is observed that the sucrose octaacetate and α-cellobiose octaacetate isomers were baseline resolved after 5 m of separation, with, once again, only 1 minute of total acquisition time. While the individual arrival time distributions for each isomer (see ESI†) displayed only a single peak (thus indicating no minor impurities being present), we envision that based on our results from Fig. 2 that minor anomeric impurities can be detected for certain protected oligosaccharides. From our presented results, we have successfully demonstrated the utility of cIMS-MS-based separations to both rapidly (1 minute total time) and sensitively (≤2% anomeric impurities detected) resolve various protected monosaccharide building block anomers as well as demonstrated the utility for isomeric mixtures of protected oligosaccharides.

## Conclusions

**Fig. 5:**
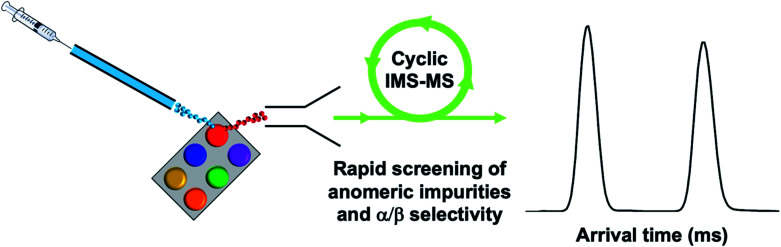
Proposed HTS platform coupling high-throughput ion introduction (*e.g.*, DESI) with high-resolution cIMS-MS separations for rapid screening of impurities and anomeric selectivity.

Herein we have presented a cIMS-MS-based workflow to serve as a first step toward the development of a high-throughput screening (HTS) platform for assessing the α/β anomeric selectivities in the carbohydrate syntheses of protected monosaccharide building blocks. Our envisioned HTS platform is highlighted in Fig. 3, where our overarching goal is to couple high-resolution cIMS-MS separations with existing automated ion introduction platforms (*e.g.*, desorption electrospray ionization, DESI, or the commercialized electrospray NanoMate by Advion).^31,44–47^ From an analytical perspective, our present results demonstrate that only 1 minute is necessary for each monosaccharide building block anomer separation and oligosaccharide isomer separation which is in good timescale agreement with the aforementioned DESI/NanoMate ionization sources (Fig. 5). We envision that acquisition times could even be lowered to 10s of seconds. Additionally, we found the limits of detection for our presented results (see ESI†) to be ∼1 nM, thus demonstrating sample quantities of <1 ng are required. Our proposed cIMS-MS HTS platform would not only enable an online and streamlined approach to assess anomer separations and corresponding selectivities but also to rapidly screen out the entire reaction landscape in complex carbohydrate syntheses. This would potentially better help facilitate reaction optimization and lead to new improvements in existing synthetic carbohydrate chemistry and the field of glycoscience as a whole.

## Conflicts of interest

There are no conflicts of interest do declare.

## Supplementary Material

RA-011-D1RA08746K-s001
