# Supplementary material for: Rapid cyclic ion mobility separations of monosaccharide building blocks as a first step toward a high-throughput reaction screening platform for carbohydrate syntheses
Source: RSC Adv. 2021 Dec 14;11(63):39742–7. doi: 10.1039/d1ra08746k (PMC9044565; doi:10.1039/d1ra08746k)
Supplement: RA-011-D1RA08746K-s001 [file RA-011-D1RA08746K-s001.pdf]

Electronic Supplementary Material (ESI) for RSC Advances.
This journal is © The Royal Society of Chemistry 2021

Electronic Supplementary Information for:

Rapid Cyclic Ion Mobility Separations of Monosaccharide Building Blocks as a First Step toward a High-Throughput Reaction Screening Platform for Carbohydrate Syntheses

Tyler L. Peterson, Gabe Nagy*

Department of Chemistry, University of Utah, 315 South 1400 East, Room 2020, Salt Lake City, Utah 84112, United States

Corresponding author: gabe.nagy@utah.edu

Additional Separation Results S2–S7

Additional separation results:

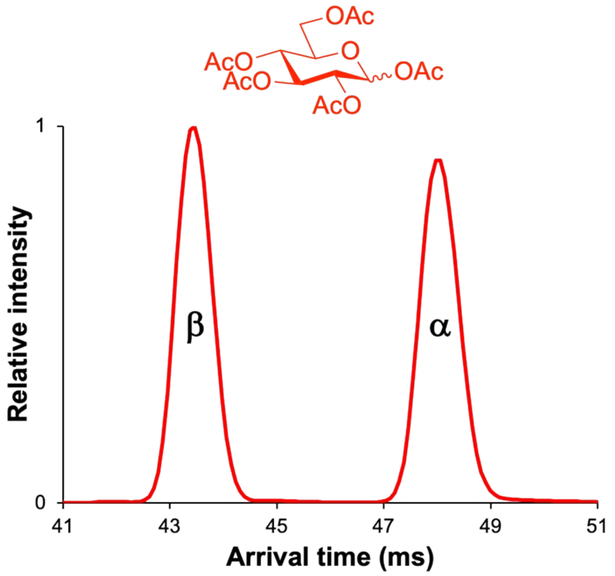

Figure S1. 5 m cIMS-MS separation of an equimolar mixture of α/β-D-glucose pentaacetate as their $[M + Na]^+$ adducts at traveling wave conditions of 450 m/s and 22 V. Each anomer was run individually (see manuscript) to assign the arrival time order.

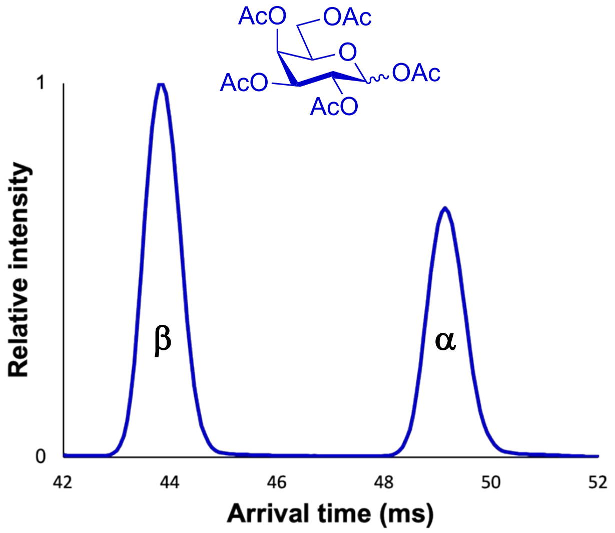

Figure S2. 5 m cIMS-MS separation of an equimolar mixture of α/β-D-galactose pentaacetate as their $[M + Na]^+$ adducts at traveling wave conditions of 450 m/s and 22 V. Each anomer was run individually (see manuscript) to assign the arrival time order.

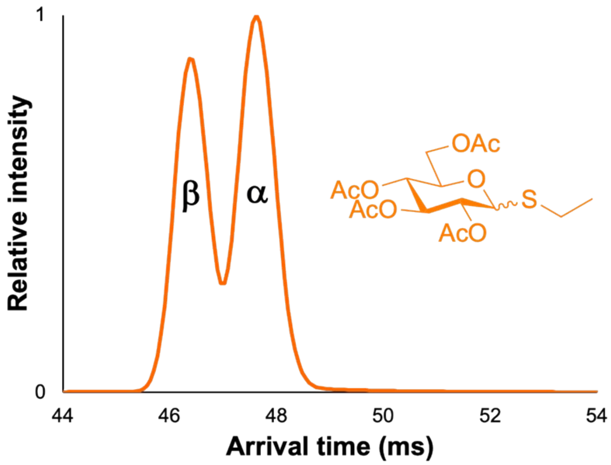

Figure S3. 5 m cIMS-MS separation of an equimolar mixture of ethyl 2,3,4,6-tetra-O-acetyl-α/β-D-thioglucopyranoside as their $[M + Na]^+$ adducts at traveling wave conditions of 450 m/s and 25 V. Each anomer was run individually (see manuscript) to assign the arrival time order.

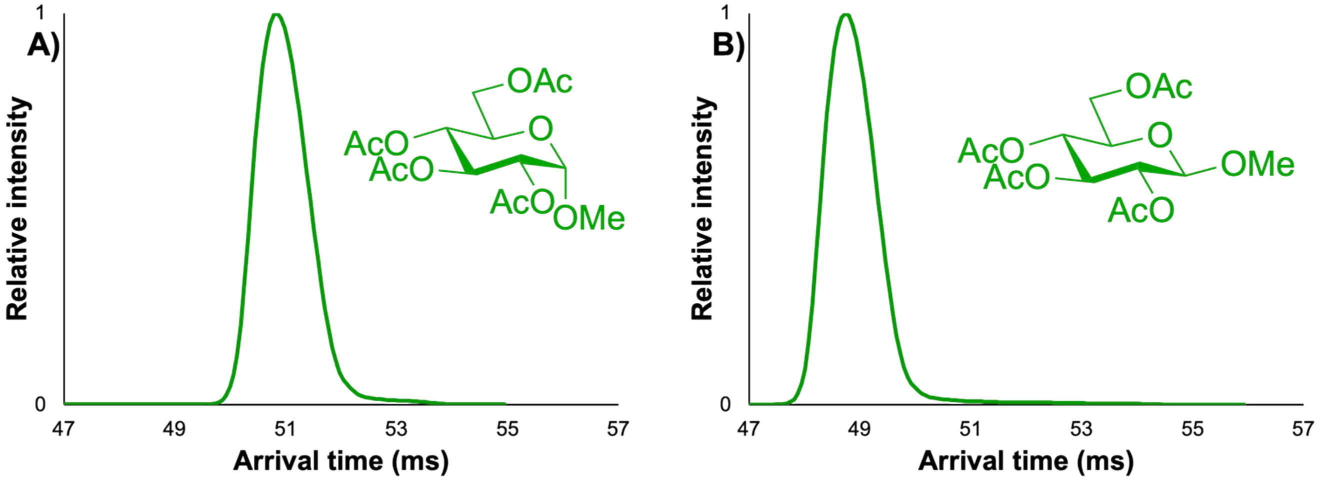

Figure S4. 5 m cIMS-MS separations of the $[M + Na]^+$ adducts for methyl 2,3,4,6-tetra-O-acetyl-α-D-glucopyranoside (A) and methyl 2,3,4,6-tetra-O-acetyl-β-D-glucopyranoside (B) at traveling wave conditions of 450 m/s and 20 V. We note no unwanted anomeric impurity was observed for each individual standard.

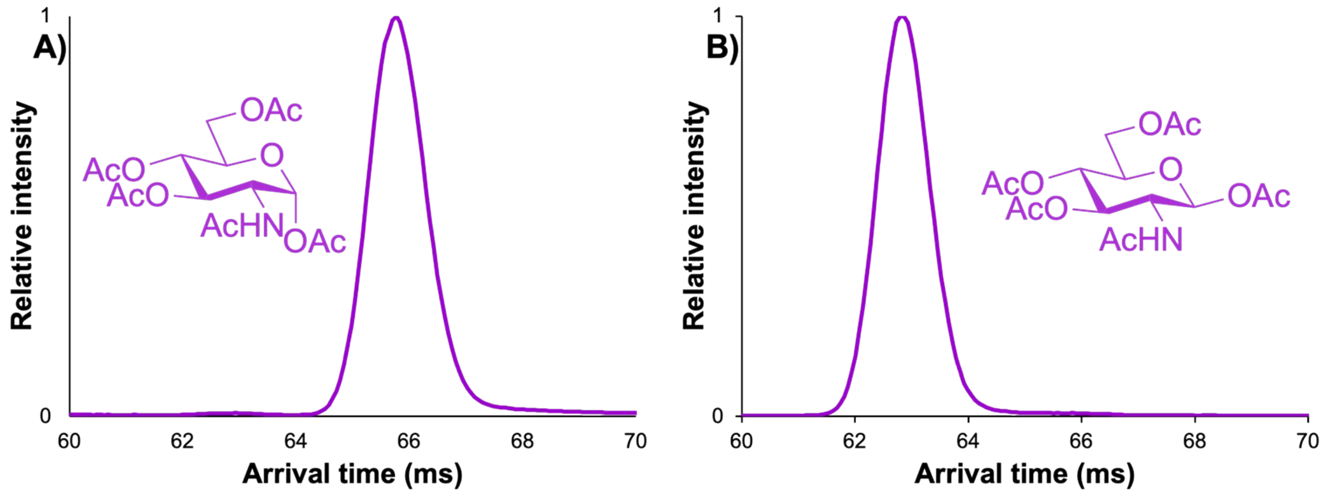

Figure S5. 5 m cIMS-MS separations of the [M + Na]+ adducts for α-D-glucosamine-pentaacetate (A) and β-D-glucosamine-pentaacetate (B) at traveling wave conditions of 375 m/s and 17 V. We note no unwanted anomeric impurity was observed for each individual standard.

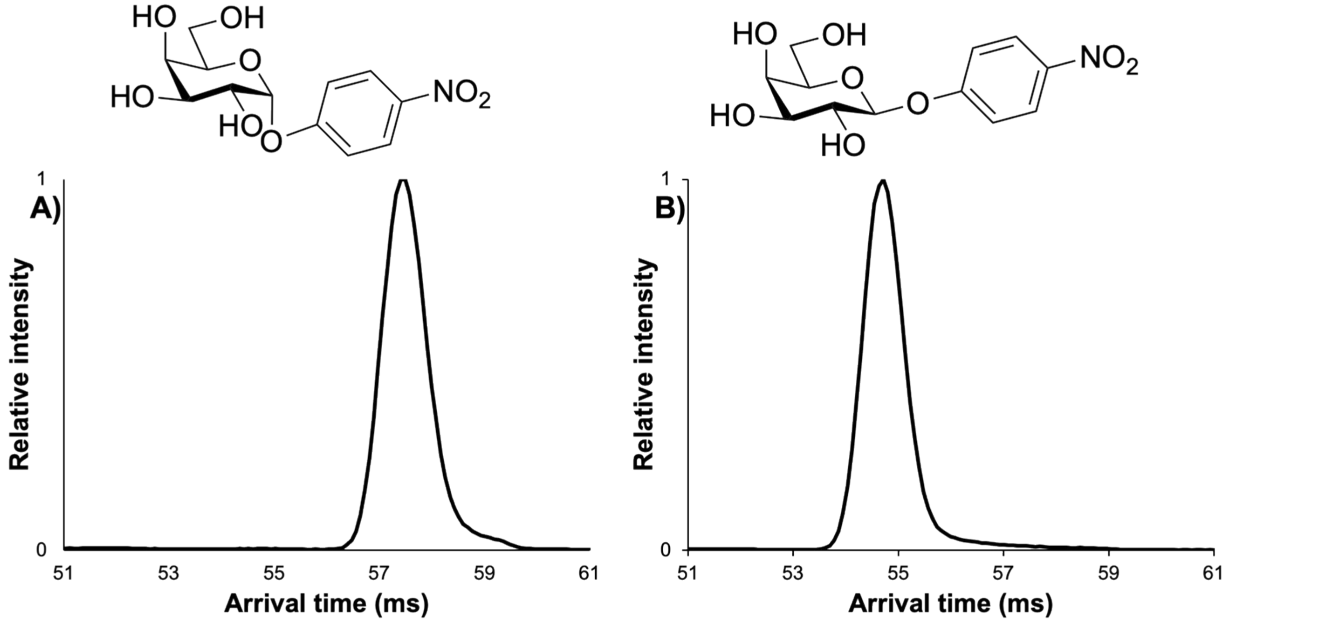

Figure S6. 5 m cIMS-MS separations of the [M + Na]+ adducts for 4-Nitrophenyl-α-D-galactopyranoside (A) and 4-Nitrophenyl-β-D-galactopyranoside (B) at traveling wave conditions of 500 m/s and 20 V. We note no unwanted anomeric impurity was observed for each individual standard.

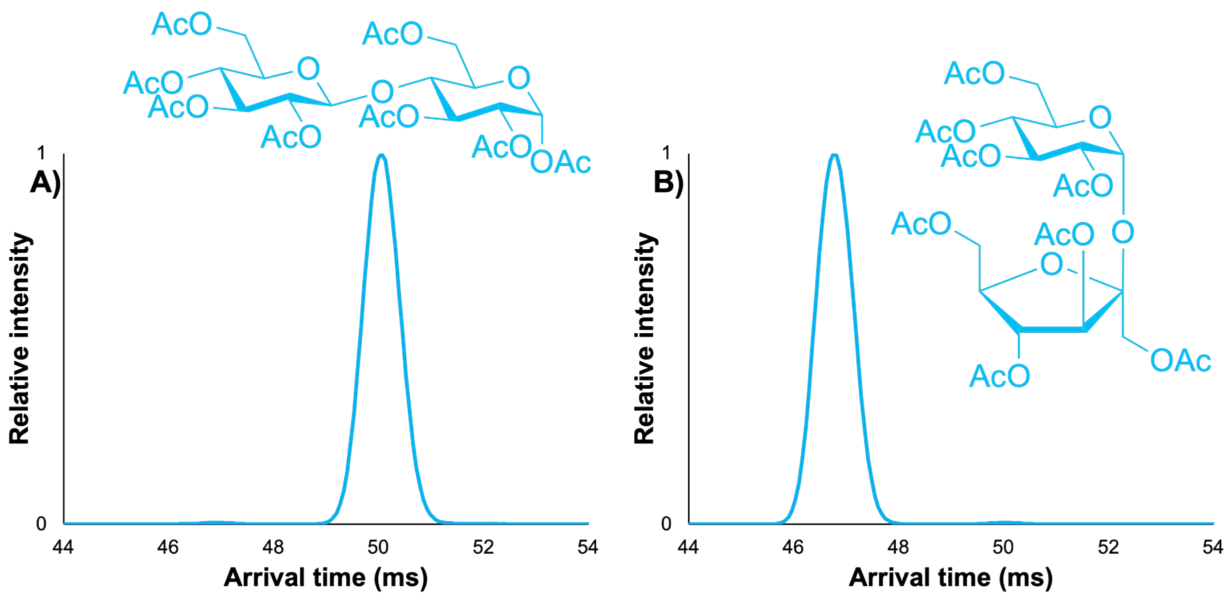

Figure S7. 5 m cIMS-MS separations of the [M + Na]$^+$ adducts for Sucrose octaacetate (A) and α-D-Cellobiose octaacetate (B) at traveling wave conditions of 350 m/s and 25 V. We note no unwanted impurity was observed for each individual standard.

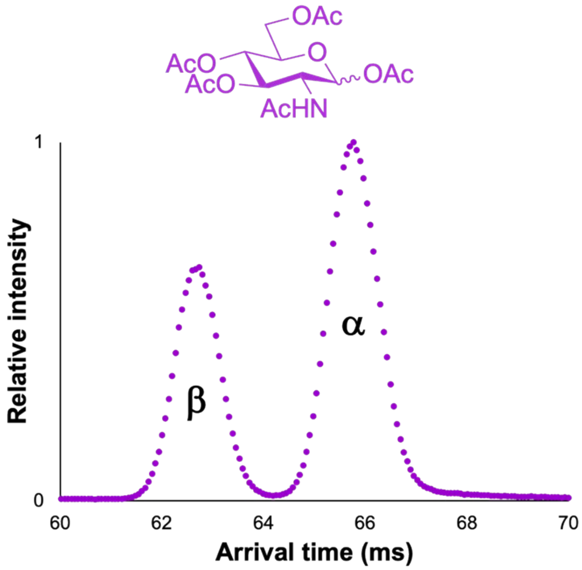

Figure S8. 5 m cIMS-MS separation of an equimolar mixture of α/β-D-glucosamine-pentaacetate as its [M + Na]$^+$ adducts at traveling wave conditions of 375 m/s and 17 V. Displayed are the 200 TOF pushes (purple dots).

We also analyzed the α/β anomers for 2,3,4,6-tetra-O-acetyl-D-glucopyranosyl trichloroacetimidate. Interestingly, the α anomer form showed signs of extremely poor ionization efficiency (as evidenced by ~100 times lower signal when run individually at the same concentration as its β anomer counterpart). This was especially evident in our results from the equimolar (50/50) mixture in Figure S9A, where the anomers are indeed resolved after 5 m, but in drastically different intensities (contrary to our other results). Additionally, the β anomer was observed to ionize with much greater efficiency than the α one as shown in Figure S9B, where the individually run α species still contained the β anomeric impurity at much higher intensity levels.

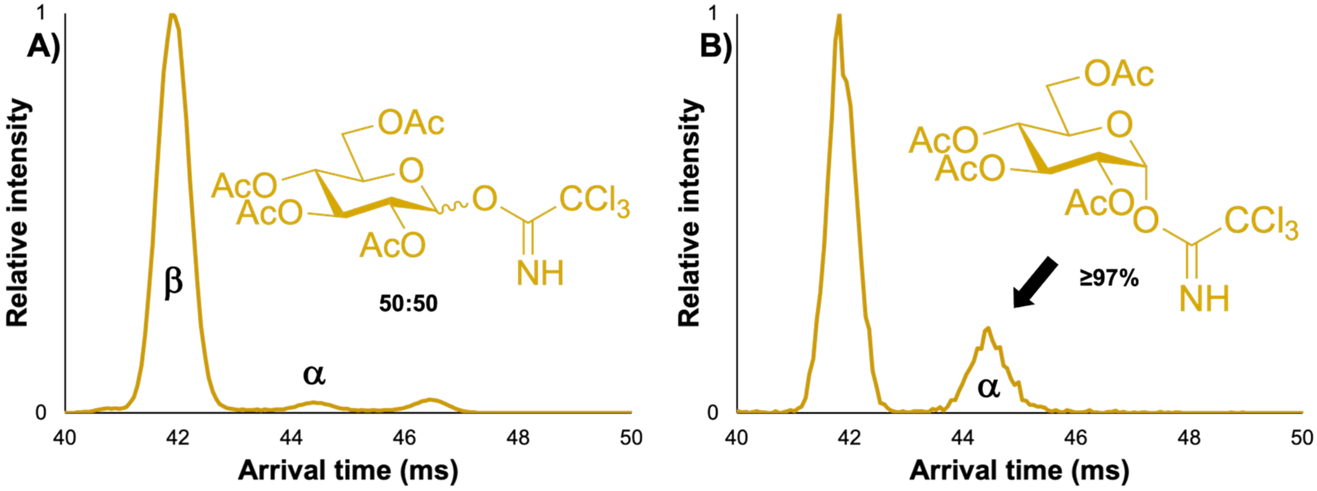

Figure S9. 5 m cIMS-MS separation of an equimolar mixture of 2,3,4,6-tetra-O-acetyl-α/β-D-glucopyranosyl trichloroacetimidate as their $[M + Na]^+$ adducts at traveling wave conditions of 450 m/s and 25 V (A) and 2,3,4,6-tetra-O-acetyl-α-D-glucopyranosyl trichloroacetimidate as its $[M + Na]^+$ adduct at traveling wave conditions of 450 m/s and 25 V (B).

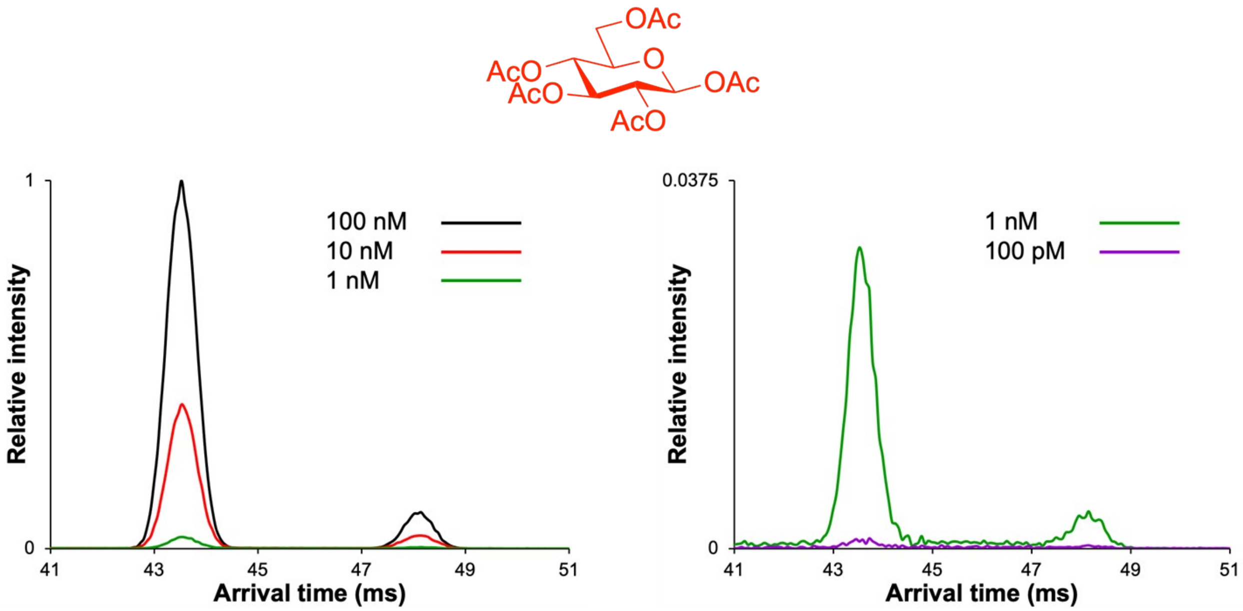

Figure S10. Arrival time distributions for the β-D-glucose pentaacetate anomer run at various concentrations as its [M + Na]+ adduct after 5 m of cIMS-MS separation at the same TW conditions as presented in the manuscript (Figure 2). We note the lower mobility peak is the minor α-anomer impurity. We observe an LOD of ~1 nM, where S/N ratios fell below 3 at concentrations of < 1 nM.
